# Elevated Resistin Gene Expression in African American Estrogen and Progesterone Receptor Negative Breast Cancer

**DOI:** 10.1371/journal.pone.0157741

**Published:** 2016-06-17

**Authors:** Karin A. Vallega, NingNing Liu, Jennifer S. Myers, Kaixian Yu, Qing-Xiang Amy Sang

**Affiliations:** 1 Department of Chemistry and Biochemistry, Florida State University, Tallahassee, Florida, United States of America; 2 Department of Statistics, Florida State University, Tallahassee, Florida, United States of America; 3 Institute of Molecular Biophysics, Florida State University, Tallahassee, Florida, United States of America; University of South Alabama, UNITED STATES

## Abstract

**Introduction:**

African American (AA) women diagnosed with breast cancer are more likely to have aggressive subtypes. Investigating differentially expressed genes between patient populations may help explain racial health disparities. Resistin, one such gene, is linked to inflammation, obesity, and breast cancer risk. Previous studies indicated that resistin expression is higher in serum and tissue of AA breast cancer patients compared to Caucasian American (CA) patients. However, resistin expression levels have not been compared between AA and CA patients in a stage- and subtype-specific context. Breast cancer prognosis and treatments vary by subtype. This work investigates differential resistin gene expression in human breast cancer tissues of specific stages, receptor subtypes, and menopause statuses in AA and CA women.

**Methods:**

Differential gene expression analysis was performed using human breast cancer gene expression data from The Cancer Genome Atlas. We performed inter-race resistin gene expression level comparisons looking at receptor status and stage-specific data between AA and CA samples. DESeq was run to test for differentially expressed resistin values.

**Results:**

Resistin RNA was higher in AA women overall, with highest values in receptor negative subtypes. Estrogen-, progesterone-, and human epidermal growth factor receptor 2- negative groups showed statistically significant elevated resistin levels in Stage I and II AA women compared to CA women. In inter-racial comparisons, AA women had significantly higher levels of resistin regardless of menopause status. In whole population comparisons, resistin expression was higher among Stage I and III estrogen receptor negative cases. In comparisons of molecular subtypes, resistin levels were significant higher in triple negative than in luminal A breast cancer.

**Conclusion:**

Resistin gene expression levels were significantly higher in receptor negative subtypes, especially estrogen receptor negative cases in AA women. Resistin may serve as an early breast cancer biomarker and possible therapeutic target for AA breast cancer.

## Introduction

According to the American Cancer Society the incidence rate of breast cancer among African American (AA) women is slightly lower than that for Caucasian American (CA) women, with 124.3 cases versus 128.1 cases per 100,000 when age adjusted to year 2000 US standard population from 2008–2012 in the United States [1]. However, AA women have a 42% higher mortality rate than CA women [1]. Growing evidence shows that both biological and non-biological factors contribute to this disparity [2]. AA women present with breast cancer at earlier ages and with more advanced stages and more aggressive histologic features [2]. In a study comparing breast cancer among AA, CA, and Hispanic women, it was shown that AA women were more likely to have tumors with worse pathological characteristics, such as larger tumor size and less differentiated cancer cells [3]. AA breast cancer patients are more likely to have disadvantageous tumor subtypes, such as estrogen receptor (ER) negative, progesterone receptor (PR) negative, and triple negative breast cancer [3–6]. AA breast cancer tissues were found to have mutations in cell cycle control components when compared to CA breast cancer tissues [7]. Another study found higher methylation rates of tumor suppressor genes and other genes involved in malignant transformation in AA breast cancers compared to CA breast cancers [8]. Our lab previously reported several genes and pathways that were differentially expressed in AA breast cancer tumors. The resistin gene had expression levels almost 5-fold greater in AA breast cancer tumors than in CA women [9].

Resistin is a member of the resistin-like molecules (RELM) family. Resistin acts as a proinflammatory factor, stimulating expression of other proinflammatory cytokines and chemokines such as interleukin 6 (IL-6) and tumor necrosis factor α (TNF-α) [10]. In numerous *in vivo* studies elevated levels of resistin have been linked to increased incidence risk of many cancers, including breast, endometrial, colorectal, lung, gastric, and esophageal cancers [10]. High levels of resistin in both serum and plasma have been linked to risk of breast cancer [11,12]. In addition, resistin expression levels were higher in breast cancer tissues than in adjacent non-malignant breast tissue [13]. High level of resistin expression correlated with increased tumor stage, size, and lymph node metastasis [13]. Furthermore, serum resistin levels were higher in postmenopausal breast cancer patients than in healthy controls, indicating that resistin is a serum biomarker for postmenopausal breast cancer [14].

Current epidemiological and experimental studies indicate that the endocrine function of adipose tissue, especially white adipose tissue, is to secrete adipocytokines such as resistin, adiponectin and leptin. Obesity and weight gain have also been directly correlated to increased breast cancer mortality [15–17]. Differences in obesity rates could also contribute to health disparities, since AA women have higher levels of obesity (56.6%) than CA women (32.8%) in the US [18]. The mechanism by which obesity increases cancer risk is not completely understood. However, possible mechanisms include changes in secretions of adipokines and cytokines and chronic subclinical inflammation [10]. Because of its inflammatory properties and role in adipose tissue, resistin is being investigated as a link between inflammation, obesity, and cancer [10].

Although our previous study found that resistin expression levels were markedly increased in AA tumors, resistin expression in different subtypes, stages, and matched non-malignant cases was not investigated. Examining gene expression by subtype is important because it is well established that phenotype and prognosis vary by subtype. Additionally, treatments are specific for certain subtypes. Therefore, we analyzed resistin gene expression levels in different subtypes. We also analyzed resistin gene expression by stage to shed light on how resistin expression might change as breast cancer progresses. In all these evaluations, we compared AA to CA cases to better understand the role of resistin in breast cancer and health disparities, using the whole-population expression data to clarify if differences were stage- and subtype-specific or race-specific. We provide evidence that resistin expression changes are subtype- and race-specific, but also associated with early stage breast cancer.

## Methods

Processed and de-identified human breast cancer patient data was downloaded from The Cancer Genome Atlas (TCGA). The de-identified patient data consisted of up to 20,531 RNA sequence-derived gene expression values and clinical characteristics such as age, cancer stage and receptor status. Python (The Python Software Foundation, http://www.python.org) and the R programming environment were used to sort the patient data for analysis. Breast cancer cases in men were excluded from the data set. Patient demographics for the study population are shown in Table 1. The p-value was obtained using Fisher’s exact test to note differences in age groups, tumor stage and type, and vital status between CA and AA women. Whole population comparisons included all women regardless of race. Receptor status and race were assigned to patient samples according to TCGA clinical data. Before age- and stage-matching, 704 CA samples and 123 AA samples were available for analysis. Within each stage, patients were subdivided by receptor status, race, menopause status, tumor molecular subtype, or disease state. The two subsets used for each comparison were then age-matched, except when comparing menopause status as this is a function of age. Molecular subtypes were assigned according to each patient’s receptor status: luminal A (ER-positive and/or PR-positive, human epidermal growth factor receptor (HER2)-negative), luminal B (ER-positive and/or PR-positive, HER2-positive), HER2 (ER-negative, PR-negative, HER2-positive), and triple negative (ER-negative, PR-negative, HER2-negative). DESeq (version 1.10.1) was run in the R statistical programming environment (version 3.1.1) to test for differential expression of resistin. DESeq assumes a negative binomial distribution for the expression levels of a gene for each condition, then uses a Fisher’s exact test type of procedure to calculate the p-value to test the significance of differential gene expression between two conditions (namely A and B). The log 2-fold change (between condition A and condition B) was calculated from the means of the resistin expression level of these two conditions. For the comparison of tumor to adjacent non-malignant tissue, a paired Wilcoxon rank-sum test was used as the matched samples came from the same patient. In these comparisons, normalized data from TCGA were used. The significance level was set to 0.05; therefore, if the p-value was less than 0.05, the gene was considered differentially expressed between these two conditions.

**Table 1:** Breast Cancer Patient Data from TCGA.

Characteristics	Caucasian American (n = 704)		African American (n = 123)		Significance
	Number	Pct.	Number	Pct.	p-value
**Age**					0.1617
<50	186	26.42%	43	34.96%	
50–64	307	43.61%	47	38.21%	
65+	211	29.97%	33	26.83%	
**Tumor Stage**					0.0166
I	202	28.69%	34	27.64%	
II	394	55.97%	72	58.54%	
III	88	12.50%	12	9.76%	
IV	20	2.84%	2	1.62%	
Other/NA	0	0.00%	3	2.44%	
**Tumor Type**					6.67E-08
Luminal A	296	42.05%	27	21.95%	
Luminal B	75	10.65%	3	2.44%	
Triple Negative	66	9.38%	24	19.51%	
HER2 Type	19	2.70%	4	3.25%	
Other/NA	248	35.22%	65	52.85%	
**Vital Status**					0.3563
Living	627	89.06%	106	86.18%	
Deceased	77	10.94%	17	13.82%	

## Results

Table 1 shows the characteristics of the breast cancer data set from TCGA. AA women had higher mortality at 13.82% compared to 10.94% for CA women. AA women also had more than double the cases of triple-negative breast cancer, with 19.51% of AA breast cancer being triple-negative compared to 9.38% for CA women. AA women also presented with breast cancer at earlier ages, with 34.96% of AA patients being under 50 years of age compared to 26.42% for CA women.

**Fig 1:**
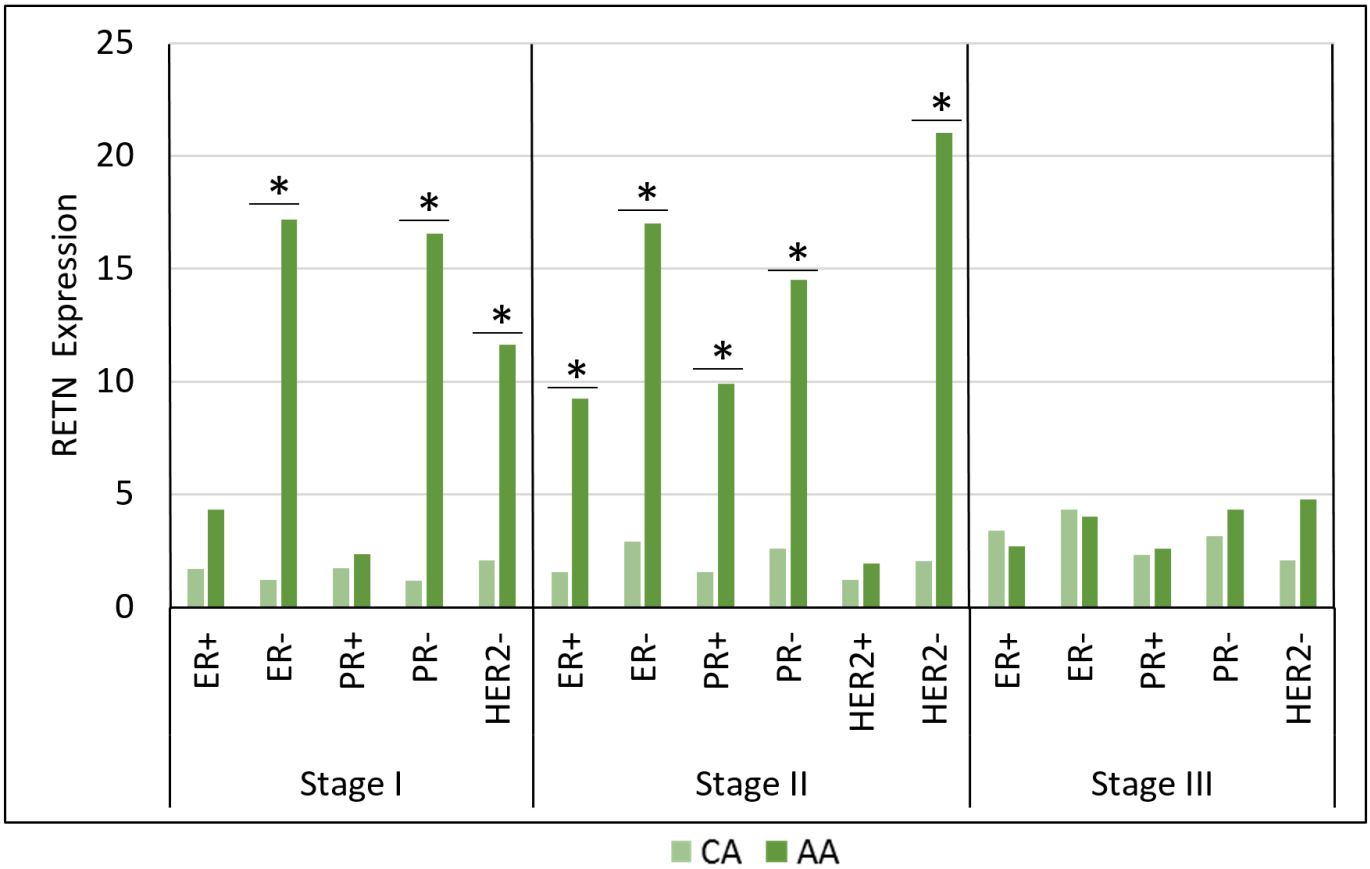
Resistin gene expression between Caucasian American and African American breast tumors by stage/receptor status.

Unfortunately, Stage IV could not be included in inter-racial comparisons due to inadequate sample sizes. A number of comparisons were significant when comparing resistin gene expression levels in CA and AA women grouped by stage and receptor status (Fig 1, S1 Table). In both Stages I and II, resistin was elevated in ER-negative AA tumors. Resistin was also associated with PR-negative status in Stages I and II. In both Stages I and II, HER2-negative AA patients showed higher levels of resistin. These results indicate that resistin is not only associated with racial disparity in breast cancer, but also with receptor negative subtypes that often carry worse prognosis.

**Fig 2:**
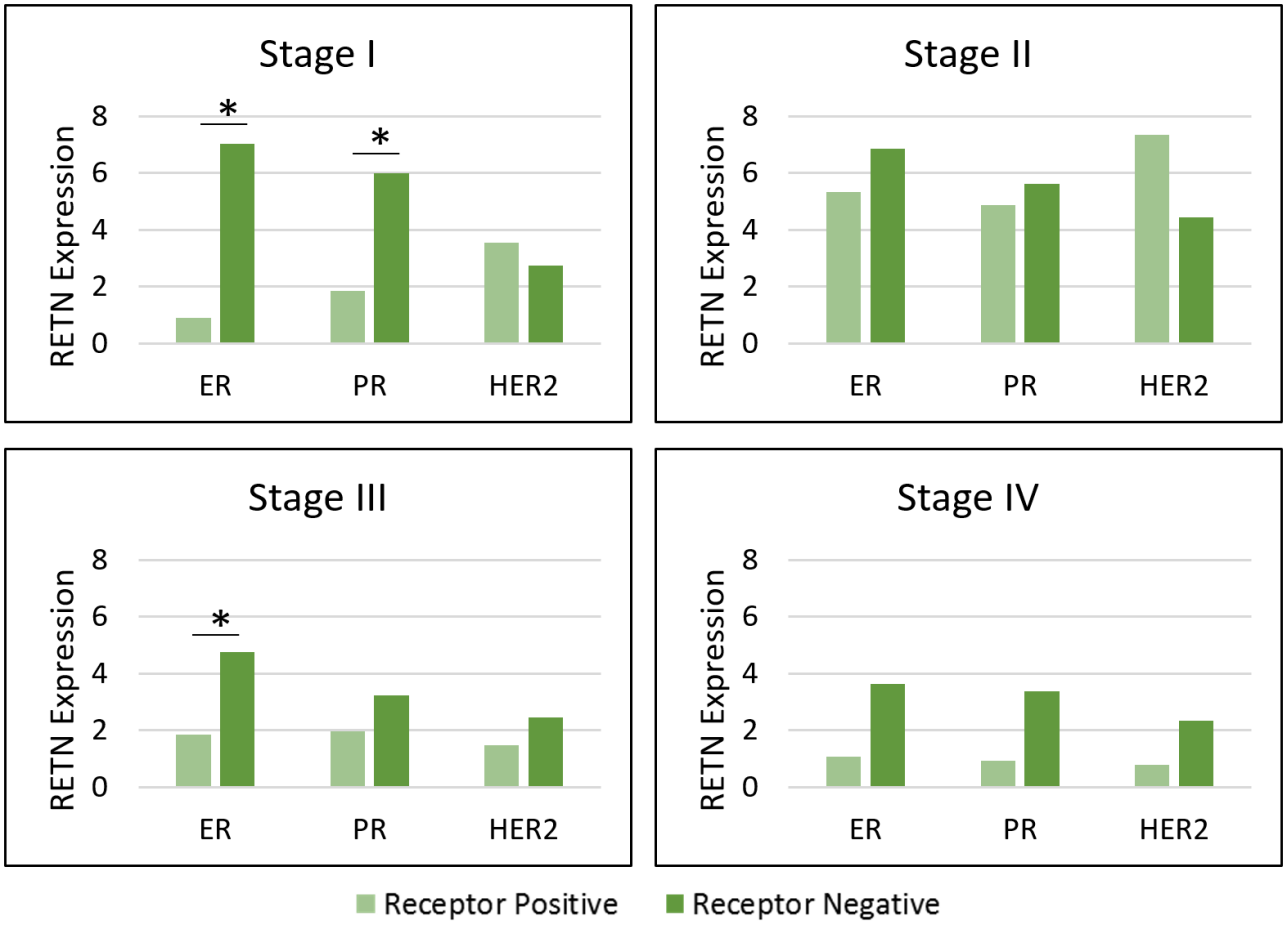
Resistin gene expression in whole population breast tumors by stage and receptor status.

To examine if resistin correlated with aggressive tumor subtypes, we divided the entire population sample set by receptor status. The ER status comparison was statistically significant in Stages I and III (Fig 2, S2 Table). The PR status comparison was also significant, but only in Stage I. Therefore, resistin was more associated with ER-negative status in Stages I and III, but only associated with PR-negative status in Stage I. However, there was also a general trend toward higher resistin expression in all ER-negative and PR-negative comparisons, even if some comparisons did not reach statistical significance. We did not see any association of resistin with HER2 receptor status in the whole population.

Next, all patients were grouped by molecular subtype according to their receptor status and compared (S4 Table). The only differentially expressed comparison was between triple negative (TNeg) breast cancer and Luminal A (LumA) breast cancer. Resistin was more highly expressed in TNeg, which follows the observed trend of higher expression in ER- and PR-negative subtypes.

**Fig 3:**
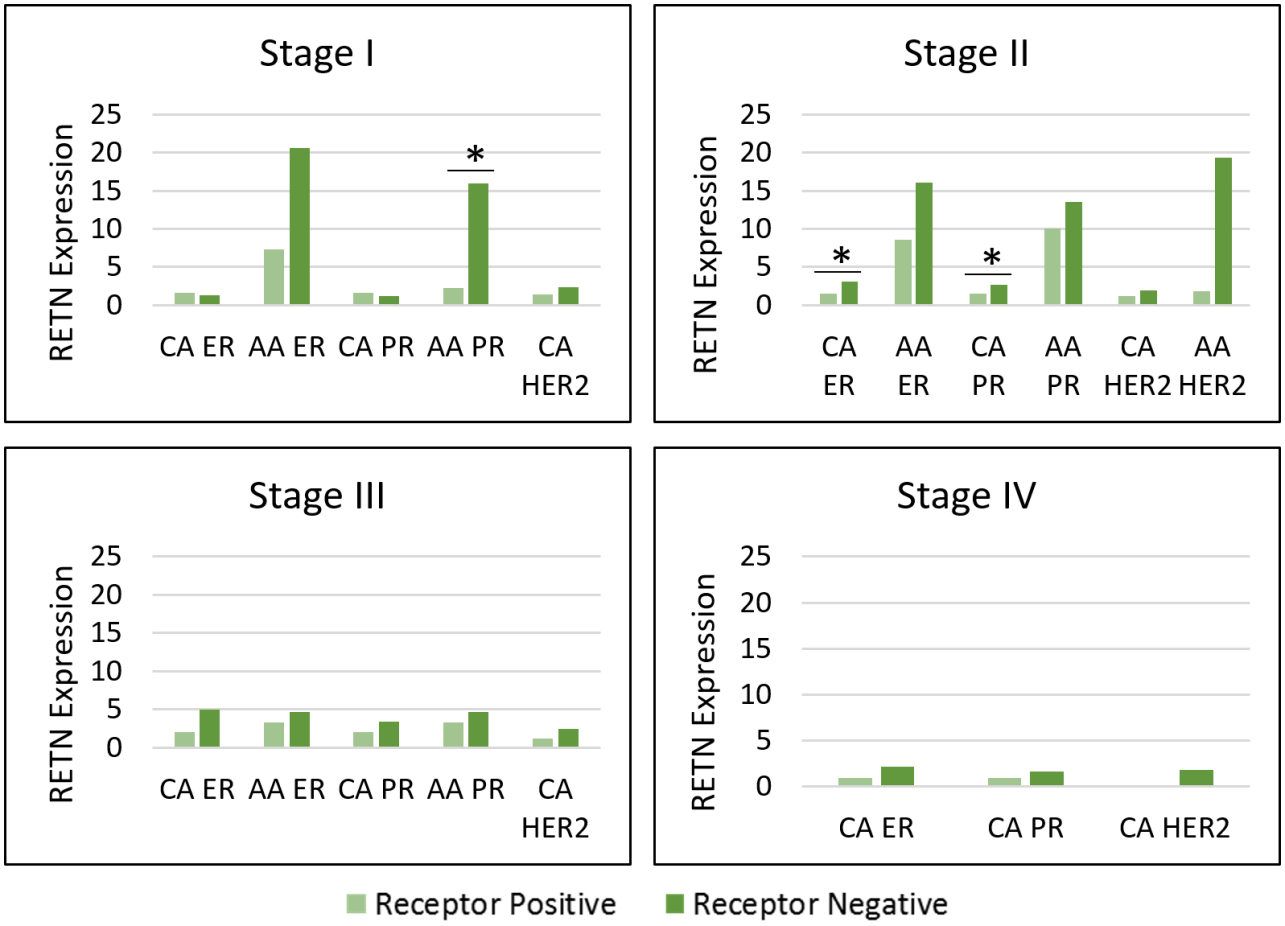
Resistin gene expression within Caucasian American and African American breast tumors by stage/receptor status.

To determine if the changes seen in resistin levels were a result of only race disparities, an intra-racial comparison was also done (Fig 3, S3 Table). Patients were again divided by breast cancer stage and then compared by receptor status within the same race. This yielded only a few significant comparisons in earlier stages in PR and ER status. Resistin was associated with PR-negative status in Stage I for AA women. Resistin was also associated with CA ER- and PR-negative status in Stage II, although the overall values are still lower for CA women. Again, in AA women resistin levels were continuously higher in receptor negative subtypes, even when these comparisons did not reach significance.

As previously noted, high levels of resistin have been linked to risk of breast cancer, particularly in post-menopausal women [14,19]. Therefore, we compared menopause status between CA and AA women (Table 2). In all Stage I and II comparisons, resistin was differentially expressed and higher in AA women regardless of menopause status. When resistin was compared within race by menopause status there were no significant results, although resistin expression tended to be higher in the post-menopausal groups (Table 3).

**Table 2:** Inter-racial comparison of resistin gene expression between Caucasian American and African American breast tumors by stage/menopause status.

Stage	Condition	Condition A	Condition B	Mean	Mean A	Mean B	Log 2 F.C.	p-value	Significance
**Stage I**	**Pre-menopause**	CA (37)	AA (9)	2.32	1.17	7.06	2.59	3.35E-03	*
	**Post-menopause**	CA (140)	AA (17)	2.79	1.82	10.79	2.57	3.02E-03	*
**Stage II**	**Pre-menopause**	CA (92)	AA (19)	3.34	2.09	9.42	2.17	6.07E-04	*
	**Post-menopause**	CA (263)	AA (42)	2.98	1.76	10.57	2.58	1.16E-07	*
**Stage III**	**Pre-menopause**	CA (21)	AA (3)	0.77	0.68	1.40	1.04	4.37E-01	
	**Post-menopause**	CA (51)	AA (6)	3.24	3.20	3.63	0.18	4.97E-01	

Patients used in the comparisons were age- and stage-matched. The numbers in parenthesis denote the number of patients used in each condition. A star for significance denotes the p-value was statistically significant. Fold change is Condition A to Condition B. Table abbreviations: F.C.–Fold change.

**Table 3:** Intra-racial comparison of resistin gene expression of Caucasian American and African American breast tumors by stage/menopause status.

Stage	Condition	Condition A	Condition B	Mean	Mean A	Mean B	Log 2 F.C.	p-value	Significance
**Stage I**	**CA**	Pre-meno. (37)	Post-meno. (140)	1.70	1.15	1.84	0.69	4.60E-01	
	**AA**	Pre-meno. (9)	Post-meno. (17)	9.13	6.59	10.47	0.67	6.33E-01	
**Stage II**	**CA**	Pre-meno. (92)	Post-meno. (263)	1.88	2.06	1.82	-0.18	6.48E-01	
	**AA**	Pre-meno. (19)	Post-meno. (42)	9.23	8.23	9.69	0.24	6.48E-01	
**Stage III**	**CA**	Pre-meno. (21)	Post-meno. (51)	2.46	0.67	3.19	2.26	5.10E-02	
	**AA**	Pre-meno. (3)	Post-meno. (6)	3.04	1.47	3.82	1.38	1.00E+00	

Patients used in the comparisons were age- and stage-matched. The numbers in parenthesis denote the number of patients used in each condition. A star for significance denotes the p-value was statistically significant. Fold change is Condition A to Condition B. Table abbreviations: CA—Caucasian American; AA—African American; F.C.—Fold change; Meno.—Menopause.

Matched non-malignant samples from non-involved tissues adjacent to the human breast cancer tissues were also obtained from TCGA. In our previous study, benign samples were not compared to tumor samples [9]. A paired Wilcoxon rank-sum test was performed to obtain p-values since the non-malignant samples were from the same patients as the tumor samples. However, none of the comparisons yielded a significant p-value (Table 4). To investigate if this was due to normal tissue being influenced by the tumor microenvironment, the same analysis was done on interleukin-1α and interleukin-1β. Again, no comparisons yielded a significant p-value (S5 Table).

**Table 4:** P-value of resistin gene expression between matched malignant and non-malignant breast samples.

Population	Condition A	Condition B	p-value
**Overall**	Non-malignant (110)	Tumor (110)	0.6548
**CA**	Non-malignant (102)	Tumor (102)	0.5607
**AA**	Non-malignant (6)	Tumor (6)	0.3613

The numbers in parenthesis denote the number of patients used in each condition. Table abbreviations: CA—Caucasian American; AA—African American.

## Discussion

In this study, we examined resistin expression levels in breast cancer of CA and AA women. We determined that while resistin is expressed higher in AA women overall, it is differentially expressed in early-stage receptor negative subtypes when compared to CA women, particularly the ER-negative subtype. Previously published studies have linked elevated levels of serum resistin to higher breast cancer risk [12] and decreased survival in breast cancer patients [13]. Although an association between resistin and AA breast cancer patients has been shown by our lab [9], to the best of our knowledge no prior study has linked resistin to AA receptor negative breast cancer subtypes such as ER- and PR-negative cancers.

ER-negative status is associated with more advanced stages of breast cancer and poorer prognosis [3]. Resistin expression levels were consistently higher in ER-negative groups when compared to ER-positive groups in both inter-racial (Fig 1) and whole population comparisons (Fig 2). Specifically, in Stages I and III, resistin expression was significantly higher in ER-negative patients in the whole population. In addition, resistin expression was higher in AA ER-negative cases than in CA ER-negative cases in Stages I and II. Resistin expression was also higher in Stage I PR-negative tumors than in PR-positive tumors in the whole population. We also compared resistin expression in molecular subtypes for the whole population (S4 Table). The only significant result was TNeg versus LumA breast tumors. Resistin expression was higher in TNeg subtype, which has higher rates among AA women than in CA women [5]. Our results show that resistin is associated with receptor negative breast cancers. With fewer treatment options currently available for TNeg patients, resistin could be studied as a possible therapeutic target.

Studies have shown that resistin is differentially expressed between CA and AA women, with AA women having higher expression [9,20]. We found resistin was differentially expressed between AA and CA women specifically in Stages I and II receptor negative cases. Resistin expression was higher in AA ER-negative, PR-negative, and HER2-negative cases (Fig 1). This suggests that resistin may contribute to racial disparities in breast cancer, or that the elevated levels of resistin may be partially responsible for AA breast cancer phenotype. A possible connection could be inflammation [10]. Resistin stimulates expression of proinflammatory cytokines, such as IL-6 [20]. AA women have higher levels of IL-6 when compared to CA women [20]. The increased IL-6 levels could be a result of the higher resistin expression in AA women. Due to resistin’s inflammatory properties and role in adipose tissue, it may be a link between inflammation, obesity, and cancer [10]. AA women have higher levels of obesity (56.6%) than CA women (32.8%) in the US [18]. A large prospective study has defined an increased risk of mortality from breast cancer, up to 2.12-fold for women with increased body mass index (BMI) compared to normal weight individuals [17]. This suggests resistin could be a biochemical link between obesity related inflammation and cancer. One limitation of our study was that we were unable to verify the link between resistin and obesity due to a lack of BMI data.

Our results also showed higher resistin expression levels in early stages of breast cancer. In inter-racial evaluations, all significant results were in Stages I and II (Fig 1). There were no significant results in Stage III. Regrettably, we were not able to look at Stage IV in inter-racial comparisons due to limited samples of Stage IV AA patients. In whole population comparison of receptor statuses, two of three significant results were in Stage I. (Fig 2). For the whole population, we were able to compare receptor statuses in Stage IV, but resistin was not differentially expressed. Lastly, in intra-racial comparisons, all significant results were again in Stages I and II (Fig 3). Another study also found higher resistin levels in recently diagnosed early breast cancer cases [21]. Although this study was limited to a small subset of women (Saudi Arabian), we observed similar results in our cohort of American women from TCGA. Because resistin is linked to inflammation and we demonstrated its association with early stage breast cancer, we hypothesize resistin may be linked to early-stage inflammation rather than late-stage metastasis.

Previous studies examining the role of resistin in menopause status of breast cancer patients have been unclear. One study found no association between breast cancer risk and increased levels of serum resistin in postmenopausal women [22]. Other groups have reported that resistin is a biomarker for postmenopausal breast cancer [14,19]. In our study, the whole population comparison showed resistin was differentially expressed and higher in postmenopausal patients compared to premenopausal patients only in Stage III, meaning levels were similar in other stages. Prior studies that found elevated resistin in postmenopausal women were comparing postmenopausal breast cancer patients with healthy postmenopausal patients. In this study, postmenopausal breast cancer patients were compared to premenopausal breast cancer patients, which may account for the similar resistin levels in most stages. When we divided our data set into AA and CA, and then compared premenopausal and postmenopausal, we found resistin expression was generally higher in postmenopausal groups, but the comparisons did not reach statistical significance. This suggests there must be other factors contributing to increased breast cancer risk in postmenopausal women. We hypothesize that race could be one such factor. Interestingly, when we compared AA to CA, we saw that AA women had higher resistin expression levels regardless of menopause status (Table 2). This also provides evidence that resistin expression changes are due to racial differences.

Another noteworthy finding was that there was no significant difference in resistin gene expression levels in paired non-malignant and tumor breast cancer data. However, since these are matched samples, non-malignant tissue is taken from the same breast cancer patient, adjacent to the malignant tumor site. Non-malignant tissue can show how resistin levels change as the cancer progresses, but does not serve as a normal control as tissue from healthy women would. Tissue adjacent to the tumor may have changed because it is part of the tumor microenvironment, in which cells have changed gene expression patterns and contribute to tumor development [20,23]. To verify this hypothesis, interleukin-1α and interleukin-1β gene expression levels were compared and no significant difference between paired non-malignant and tumor samples was observed. Like resistin, interleukin-1 is produced mainly by immune cells in the adipose tissue [24]. Interleukin-1 is elevated in tumors with worse prognosis, and high interleukin-1β levels have been reported in breast cancer tumors [25]. These results suggest that similar gene expression levels are due to the paired non-malignant sample being part of the tumor microenvironment. Therefore, a limitation of this study was lack of healthy controls. In addition, intra-tumor heterogeneity would have to be considered to determine if resistin has potential as a tissue biomarker. A single tumor could have multiple loci with varying genomic alterations [26]. Therefore, even in patients with the same tumor type, resistin expression levels might vary. Moreover, different cells in the tumor microenvironment can produce resistin, therefore varying numbers of adipocytes and immune cells present in the tumor microenvironment could account for the changes in resistin gene expression levels. More data are needed to determine if resistin could serve as a diagnostic breast cancer biomarker.

## Conclusion

In conclusion, we confirmed higher resistin expression in AA women and discovered that resistin is associated with ER-, PR-, HER2-negative and triple negative subtypes, which are more aggressive breast cancer subtypes with limited treatment options. Targeting resistin may be a novel therapeutic option. Because resistin gene expression is higher in early stages (I and II), especially in AA breast cancer patients, it could serve as an early detection biomarker. Early and accurate diagnosis is essential to successful treatment. However, more research is needed to clarify resistin’s role in receptor negative breast cancer to translate this basic science research to future clinical applications.

## Supporting Information

S1 Table Resistin gene expression between Caucasian American and African American breast tumors by Stage/receptor status. Patients used in the comparisons were age- and stage-matched. The numbers in parenthesis denote the number of patients used in each condition. A star for significance denotes the p-value was statistically significant. Fold change is Condition A to Condition B. Table abbreviations: Cond.—Condition; F.C.—Fold change; CA—Caucasian American; AA—African American; ER—Estrogen Receptor; PR—Progesterone Receptor; HER2 —Human Epidermal Growth Factor Receptor 2.
(DOCX)

S2 Table Resistin gene expression in whole population breast tumors by stage and receptor status. Patients used in the comparisons were age- and stage-matched. The numbers in parenthesis denote the number of patients used in each condition. A star for significance denotes the p-value was statistically significant. Fold change is Condition A to Condition B. Table abbreviations: Cond.—Condition; F.C.—Fold change; Meno.—Menopause; ER—Estrogen Receptor; PR—Progesterone Receptor; HER2 —Human Epidermal Growth Factor Receptor 2.
(DOCX)

S3 Table Resistin gene expression within Caucasian American and African American breast tumors by stage/receptor status. Patients used in the comparisons were age- and stage-matched. The numbers in parenthesis denote the number of patients used in each condition. A star for significance denotes the p-value was statistically significant. Fold change is Condition A to Condition B. Table abbreviations: Cond.—Condition; F.C.—Fold change; CA—Caucasian American; AA—African American; ER—Estrogen Receptor; PR—Progesterone Receptor; HER2 —Human Epidermal Growth Factor Receptor 2.
(DOCX)

S4 Table Overall comparison between molecular subtypes. Patients used in the comparisons were age- and stage-matched. The numbers in parenthesis denote the number of patients used in each condition. A star for significance denotes the p-value was statistically significant. Fold change is Condition A to Condition B. Table abbreviations: Cond.–Condition; F.C.—Fold change; TNeg—Triple negative; LumA—Luminal A; LumB—Luminal B; HER2t —HER2 type; ER—Estrogen Receptor; PR—Progesterone Receptor; HER2 —Human Epidermal Growth Factor Receptor 2.
(DOCX)

S5 Table P-value of interleukin-1 gene expression between matched malignant and non-malignant breast samples. The numbers in parenthesis denote the number of patients used in each condition. Table abbreviations: CA—Caucasian American; AA—African American.
(DOCX)

## Abbreviations

AA: African American
CA: Caucasian American
ER: Estrogen receptor
PR: Progesterone receptor
HER2: Human epidermal growth factor receptor 2
BMI: Body mass index
ADSF: Adipocyte secreted factor
FIZZ-3: Found in inflammatory zone 3
RELM: Resistin-like molecules
IL-6: Interleukin 6
TNF-α: Tumor necrosis factor α
TCGA: The Cancer Genome Atlas
TNeg: Triple negative
LumA: Luminal A
LumB: Luminal B
HER2t: HER2 type
