# Supplementary material for: Elevated Resistin Gene Expression in African American Estrogen and Progesterone Receptor Negative Breast Cancer
Source: PLoS One. 2016 Jun 17;11(6):e0157741. doi: 10.1371/journal.pone.0157741 (PMC4912107; doi:10.1371/journal.pone.0157741)
Supplement: S1 Table — Patients used in the comparisons were age- and stage-matched. The numbers in parenthesis denote the number of patients used in each condition. A star for significance denotes the p-value was statistically significant. Fold change is Condition A to Condition B. Table abbreviations: Cond.—Condition; F.C.—Fold change; CA—Caucasian American; AA—African American; ER—Estrogen Receptor; PR—Progesterone Receptor; HER2 —Human Epidermal Growth Factor Receptor 2. (DOCX) [file pone.0157741.s001.docx]

| **Stage** | **Cond. A** | **Cond. B** | **Mean** | **Mean A** | **Mean B** | **Log 2 F.C.** | **p-value** | **Significance** |
| --- | --- | --- | --- | --- | --- | --- | --- | --- |
| **Stage I** | CA ER+ (159) | AA ER+ (18) | 1.97 | 1.70 | 4.32 | 1.34 | 1.59E-01 |  |
|  | CA ER- (32) | AA ER- (16) | 6.55 | 1.23 | 17.18 | 3.80 | 8.81E-05 | * |
|  | CA PR+ (141) | AA PR+ (15) | 1.81 | 1.75 | 2.35 | 0.43 | 6.86E-01 |  |
|  | CA PR- (48) | AA PR- (19) | 5.55 | 1.20 | 16.56 | 3.79 | 2.46E-06 | * |
|  | CA HER2- (113) | AA HER2- (13) | 3.06 | 2.08 | 11.63 | 2.49 | 1.20E-02 | * |
| **Stage II** | CA ER+ (287) | AA ER+ (44) | 2.57 | 1.55 | 9.25 | 2.58 | 1.24E-08 | * |
|  | CA ER- (85) | AA ER- (29) | 6.50 | 2.91 | 17.00 | 2.54 | 2.38E-04 | * |
|  | CA PR+ (248) | AA PR+ (35) | 2.58 | 1.56 | 9.90 | 2.67 | 4.26E-03 | * |
|  | CA PR- (100) | AA PR- (38) | 5.89 | 2.59 | 14.50 | 2.49 | 9.49E-05 | * |
|  | CA HER+ (66) | AA HER2+ (6) | 1.27 | 1.21 | 1.94 | 0.68 | 3.10E-01 |  |
|  | CA HER2- (160) | AA HER2- (32) | 5.20 | 2.04 | 21.02 | 3.37 | 1.89E-08 | * |
| **Stage III** | CA ER+ (20) | AA ER+ (7) | 3.21 | 3.39 | 2.71 | -0.32 | 9.39E-01 |  |
|  | CA ER- (12) | AA ER- (5) | 4.23 | 4.32 | 4.03 | -0.10 | 7.51E-01 |  |
|  | CA PR+ (25) | AA PR+ (7) | 2.39 | 2.34 | 2.60 | 0.15 | 8.56E-01 |  |
|  | CA PR- (23) | AA PR- (5) | 3.37 | 3.16 | 4.33 | 0.45 | 4.89E-01 |  |
|  | CA HER2- (48) | AA HER2- (7) | 2.43 | 2.09 | 4.78 | 1.19 | 1.19E-01 |  |
