# Supplementary material for: Elevated Resistin Gene Expression in African American Estrogen and Progesterone Receptor Negative Breast Cancer
Source: PLoS One. 2016 Jun 17;11(6):e0157741. doi: 10.1371/journal.pone.0157741 (PMC4912107; doi:10.1371/journal.pone.0157741)
Supplement: S2 Table — Patients used in the comparisons were age- and stage-matched. The numbers in parenthesis denote the number of patients used in each condition. A star for significance denotes the p-value was statistically significant. Fold change is Condition A to Condition B. Table abbreviations: Cond.—Condition; F.C.—Fold change; Meno.—Menopause; ER—Estrogen Receptor; PR—Progesterone Receptor; HER2 —Human Epidermal Growth Factor Receptor 2. (DOCX) [file pone.0157741.s002.docx]

| **Stage** | **Cond. A** | **Cond. B** | **Mean** | **Mean A** | **Mean B** | **Log 2 F.C.** | **p-value** | **Significance** |
| --- | --- | --- | --- | --- | --- | --- | --- | --- |
| **Stage I** | Pre-meno. (49) | Post-meno. (174) | 2.69 | 2.19 | 2.83 | 0.37 | 6.40E-01 |  |
|  | ER+ (80) | ER- (50) | 3.26 | 0.91 | 7.03 | 2.96 | 1.30E-04 | * |
|  | HER2+ (25) | HER2- (117) | 2.87 | 3.55 | 2.73 | -0.38 | 5.09E-01 |  |
|  | PR+ (87) | PR- (70) | 3.70 | 1.84 | 6.00 | 1.71 | 2.00E-02 | * |
| **Stage II** | Pre-meno. (131) | Post-meno. (375) | 4.50 | 4.22 | 4.60 | 0.12 | 8.64E-01 |  |
|  | ER+ (243) | ER- (137) | 5.88 | 5.33 | 6.86 | 0.37 | 4.42E-01 |  |
|  | HER2+ (100) | HER2- (219) | 5.35 | 7.35 | 4.44 | -0.73 | 2.77E-01 |  |
|  | PR+ (258) | PR- (189) | 5.19 | 4.87 | 5.62 | 0.21 | 6.28E-01 |  |
| **Stage III** | Pre-meno. (27) | Post-meno. (68) | 2.34 | 0.75 | 2.98 | 1.99 | 3.96E-02 | * |
|  | ER+ (97) | ER- (19) | 2.33 | 1.86 | 4.74 | 1.35 | 4.54E-02 | * |
|  | HER2+ (13) | HER2- (44) | 2.22 | 1.46 | 2.44 | 0.74 | 5.57E-01 |  |
|  | PR+ (82) | PR- (35) | 2.33 | 1.95 | 3.22 | 0.72 | 2.07E-01 |  |
| **Stage IV** | Pre-meno. (4) | Post-meno. (23) | 1.49 | 3.94 | 1.06 | -1.89 | 2.87E-01 |  |
|  | ER+ (12) | ER- (9) | 2.15 | 1.06 | 3.62 | 1.77 | 2.37E-01 |  |
|  | HER2+ (7) | HER2- (16) | 1.87 | 0.79 | 2.34 | 1.56 | 4.70E-01 |  |
|  | PR+ (6) | PR- (6) | 2.15 | 0.93 | 3.37 | 1.86 | 8.31E-01 |  |
