# Supplementary material for: Elevated Resistin Gene Expression in African American Estrogen and Progesterone Receptor Negative Breast Cancer
Source: PLoS One. 2016 Jun 17;11(6):e0157741. doi: 10.1371/journal.pone.0157741 (PMC4912107; doi:10.1371/journal.pone.0157741)
Supplement: S3 Table — Patients used in the comparisons were age- and stage-matched. The numbers in parenthesis denote the number of patients used in each condition. A star for significance denotes the p-value was statistically significant. Fold change is Condition A to Condition B. Table abbreviations: Cond.—Condition; F.C.—Fold change; CA—Caucasian American; AA—African American; ER—Estrogen Receptor; PR—Progesterone Receptor; HER2 —Human Epidermal Growth Factor Receptor 2. (DOCX) [file pone.0157741.s003.docx]

| **Stage** | **Cond. A** | **Cond. B** | **Mean** | **Mean A** | **Mean B** | **Log 2 F.C.** | **p-value** | **Significance** |
| --- | --- | --- | --- | --- | --- | --- | --- | --- |
| **Stage I** | CA ER+ (80) | CA ER- (32) | 1.54 | 1.65 | 1.29 | -0.36 | 9.06E-01 |  |
|  | AA ER+ (4) | AA ER- (4) | 13.97 | 7.33 | 20.60 | 1.49 | 1.88E-01 |  |
|  | CA PR+ (90) | CA PR- (48) | 1.48 | 1.62 | 1.21 | -0.43 | 5.52E-01 |  |
|  | AA PR+ (15) | AA PR- (19) | 9.93 | 2.24 | 16.00 | 2.84 | 1.48E-03 | * |
|  | CA HER2+ (20) | CA HER2- (59) | 2.12 | 1.42 | 2.36 | 0.73 | 6.45E-01 |  |
| **Stage II** | CA ER+ (281) | CA ER- (85) | 1.91 | 1.56 | 3.07 | 0.98 | 3.74E-03 | * |
|  | AA ER+ (42) | AA ER- (29) | 11.63 | 8.59 | 16.03 | 0.90 | 1.32E-01 |  |
|  | CA PR+ (243) | CA PR- (121) | 1.92 | 1.56 | 2.65 | 0.77 | 1.44E-02 | * |
|  | AA PR+ (17) | AA PR- (17) | 11.81 | 10.05 | 13.56 | 0.43 | 4.54E-01 |  |
|  | CA HER2+ (63) | CA HER2- (149) | 1.70 | 1.24 | 1.90 | 0.61 | 1.98E-01 |  |
|  | AA HER2+ (6) | AA HER2- (28) | 16.26 | 1.84 | 19.35 | 3.39 | 6.66E-02 |  |
| **Stage III** | CA ER+ (60) | CA ER- (12) | 2.49 | 1.99 | 4.96 | 1.32 | 1.64E-01 |  |
|  | AA ER+ (5) | AA ER- (5) | 3.98 | 3.33 | 4.64 | 0.48 | 8.64E-01 |  |
|  | CA PR+ (63) | CA PR- (23) | 2.39 | 2.03 | 3.38 | 0.74 | 3.89E-01 |  |
|  | AA PR+ (5) | AA PR- (5) | 3.98 | 3.33 | 4.64 | 0.48 | 8.64E-01 |  |
|  | CA HER2+ (8) | CA HER2- (35) | 2.24 | 1.20 | 2.48 | 1.05 | 6.31E-01 |  |
| **Stage IV** | CA ER+ (10) | CA ER- (5) | 1.36 | 0.95 | 2.18 | 1.20 | 6.40E-01 |  |
|  | CA PR+ (8) | CA PR- (8) | 1.30 | 0.98 | 1.62 | 0.73 | 8.15E-01 |  |
|  | CA HER2+ (4) | CA HER2- (6) | 1.19 | 0.21 | 1.84 | 3.12 | 9.64E-01 |  |
