# Supplementary material for: Elevated Resistin Gene Expression in African American Estrogen and Progesterone Receptor Negative Breast Cancer
Source: PLoS One. 2016 Jun 17;11(6):e0157741. doi: 10.1371/journal.pone.0157741 (PMC4912107; doi:10.1371/journal.pone.0157741)
Supplement: S4 Table — Patients used in the comparisons were age- and stage-matched. The numbers in parenthesis denote the number of patients used in each condition. A star for significance denotes the p-value was statistically significant. Fold change is Condition A to Condition B. Table abbreviations: Cond.–Condition; F.C.—Fold change; TNeg—Triple negative; LumA—Luminal A; LumB—Luminal B; HER2t —HER2 type; ER—Estrogen Receptor; PR—Progesterone Receptor; HER2 —Human Epidermal Growth Factor Receptor 2. (DOCX) [file pone.0157741.s004.docx]

| **Cond. A** | **Cond. B** | **Mean** | **Mean A** | **Mean B** | **Log 2 F.C.** | **p-value** | **Significance** |
| --- | --- | --- | --- | --- | --- | --- | --- |
| TNeg (43) | LumA (43) | 3.46 | 4.88 | 2.03 | -1.26 | 2.37E-02 | * |
| LumB (38) | TNeg (38) | 11.60 | 15.11 | 8.08 | -0.90 | 5.43E-01 |  |
| LumB (110) | LumA (261) | 3.84 | 6.95 | 2.53 | -1.46 | 8.37E-02 |  |
| HER2t (34) | TNeg (93) | 7.21 | 2.86 | 8.80 | 1.62 | 1.05E-01 |  |
| LumA (187) | HER2t (34) | 2.36 | 2.26 | 2.91 | 0.36 | 2.81E-01 |  |
| LumB (97) | HER2t (34) | 3.94 | 4.31 | 2.90 | -0.57 | 1.00E+00 |  |
