# Supplementary material for: Elevated Resistin Gene Expression in African American Estrogen and Progesterone Receptor Negative Breast Cancer
Source: PLoS One. 2016 Jun 17;11(6):e0157741. doi: 10.1371/journal.pone.0157741 (PMC4912107; doi:10.1371/journal.pone.0157741)
Supplement: S5 Table — The numbers in parenthesis denote the number of patients used in each condition. Table abbreviations: CA—Caucasian American; AA—African American. (DOCX) [file pone.0157741.s005.docx]

| **Gene** | **Population** | **Condition A** | **Condition B** | **p-value** |
| --- | --- | --- | --- | --- |
| **Interleukin-1α** | **Overall** | Non-malignant (110) | Tumor (110) | 0.2370 |
|  | **CA** | Non-malignant (102) | Tumor (102) | 0.2458 |
|  | **AA** | Non-malignant (6) | Tumor (6) | 1.0000 |
| **Interleukin-1β** | **Overall** | Non-malignant (110) | Tumor (110) | 0.0615 |
|  | **CA** | Non-malignant (102) | Tumor (102) | 0.0951 |
|  | **AA** | Non-malignant (6) | Tumor (6) | 0.3125 |
